# Menstrual health and school absenteeism among adolescent girls in Uganda (MENISCUS): a feasibility study

**DOI:** 10.1186/s12905-017-0502-z

**Published:** 2018-01-03

**Authors:** George Miiro, Rwamahe Rutakumwa, Jessica Nakiyingi-Miiro, Kevin Nakuya, Saidat Musoke, Juliet Namakula, Suzanna Francis, Belen Torondel, Lorna J. Gibson, David A. Ross, Helen A. Weiss

**Affiliations:** 1 Uganda Virus Research Institute, Entebbe, Uganda; 2 Medical Research Council, Uganda Virus Research Institute, Entebbe, Uganda; 3 London School of Hygiene and Tropical Medicine, London, UK; 4 Department of Maternal, Newborn, Child and Adolescent Health, World Health Organization, Geneva, Switzerland; 5 MRC Tropical Epidemiology Group, London School of Hygiene & Tropical Medicine, Keppel Street, London, WC1E 7HT UK

**Keywords:** Menstrual health, Adolescence, Menstrual knowledge, School girls, School absenteeism, School attendance

## Abstract

**Background:**

Management of menstruation can present substantial challenges to girls in low-income settings. In preparation for a menstrual hygiene intervention to reduce school absenteeism in Uganda, this study aimed to investigate menstruation management practices, barriers and facilitators, and the influence of menstruation on school absenteeism among secondary school students in a peri-urban district of Uganda.

**Methods:**

Qualitative and quantitative studies were conducted among consenting girls and boys aged 14–17 years in four secondary schools in Entebbe sub-District, Uganda. Methods included group and in-depth interviews with students, a quantitative cross-sectional questionnaire, a prospectively self-completed menstrual diary, key informant interviews with policy makers, and observations of school water, sanitation and hygiene facilities. Multiple logistic regression was used to assess factors associated with school absenteeism during the most recent menstrual period.

**Results:**

Girls reported substantial embarrassment and fear of teasing related to menstruation in the qualitative interviews, and said that this, together with menstrual pain and lack of effective materials for menstrual hygiene management, led to school absenteeism. All policy makers interviewed reported poverty and menstruation as the key factors associated with school attendance.

The 352 girls with questionnaire data had a median age of 16 (inter-quartile range (IQR) = 15,16) years, with median age at menarche of 13 (IQR = 13,14) years. Of these, 64 girls (18.7%) reported having stained their clothes and 69 (19.7%) reported missing at least 1 day of school, during their most recent period. Missing school during the most recent period was associated with physical symptoms (headache (odds ratio (OR) = 2.15, 95%CI:1.20, 3.86), stomach pain (OR = 1.89, 95%CI:0.89, 4.04), back pain (OR = 1.75, 95%CI:0.97, 3.14), and with changing protection 4 or more times per 24 h period (OR = 2.08, 95%CI:1.06, 4.10). In the diary sub-study among 40 girls, school absence was reported on 28% of period-days, compared with 7% of non-period days (adjusted odds ratio = 5.99, 95%CI:4.4, 8.2; *p* < 0.001).

**Conclusion:**

In this peri-urban Ugandan population, menstruation was strongly associated with school attendance. Evaluation of a menstrual management intervention that address both psychosocial (e.g. self-confidence, attitudes) and physical (e.g. management of pain, use of adequate menstrual hygiene materials, improved water and sanitation facilities) aspects of menstruation are needed.

## Background

Poor management of menstruation affects many girls globally, and especially in low- and middle-income countries (LMIC) [1, 2]. Challenges associated with effective menstrual hygiene management (MHM) include lack of access to clean, effective absorbents; inadequate facilities to change, clean and dispose of absorbents; lack of access to soap and water; and lack of privacy [2–6]. In addition, inadequate social support and presence of taboos can lead to psychosocial consequences of menstruation including shame, fear, anxiety and distraction [2–6]. These can potentially affect girls’ ability to thrive and succeed within the school environment [7]. In Uganda, the Government is prioritizing improvement of MHM among girls and women, for example by the launch of the Menstrual Hygiene Charter in 2015, in which the government and civil society organizations committed they would work together to promote MHM [8].

Systematic reviews show a lack of rigorous evidence for the effect of poor MHM on health and social outcomes [9], and for the effectiveness of MHM interventions to improve education and psychosocial outcomes [10]. The reviews found some evidence that poor MHM has been found to be associated with an increased risk of reproductive tract infections [9], and that interventions may improve school attendance [10], but the quantity and quality of evidence are sparse [11–14]. Limitations of the few existing studies include small study size and challenges in assessing educational outcomes (due, for example, to inaccuracy of school registers or comparability of educational outcomes across schools, and problems in detecting school dropout), and varying definitions of MHM practices. The lack of a sufficient evidence-base on the effectiveness of MHM interventions has been highlighted by the “MHM in Ten” group, which represents United Nations agencies, non-governmental agencies, academics and stakeholders. In 2014 this group identified priorities for improving MHM by 2024 [1]. The first priority identified was to expand the evidence on the health and educational impacts of inadequate MHM, and to identify effective and cost-effective interventions to improve MHM in schools [1].

The objectives of this paper are to describe results of a mixed-methods study among peri-urban secondary school students in Wakiso District, Uganda to understand i) menstruation (patterns, symptoms, management practices, knowledge and attitudes), ii) the influence of menstruation on school attendance, and iii) methods to estimate school attendance. The results will be used to design a comprehensive MHM programme for secondary school girls in Wakiso District.

## Methods

### Study setting

The study was conducted in Entebbe sub-district, a peri-urban area in Wakiso district, Uganda. The sub-district contains 13 registered secondary schools, including three government-sponsored (public) schools. Prior to initiating the research, a stakeholders meeting was held with the Entebbe Municipality authorities in the education and health sectors, to select the schools and discuss the study purpose, objectives, procedures and duration. Four schools were purposively selected (one public Universal Secondary Education (USE); one public non-USE); and two private schools (one high socio-economic status (SES), and one low SES). The study was conducted among students in secondary school years (Forms) 2 and 3 (predominantly age 15–16 years) in these four secondary schools from October 2015 to August 2016.

Data presented in this paper are part of a larger study among both girls and boys, which focused on both menstrual practices and safe male circumcision (MENISCUS: Menstrual Hygiene and Safe Male Circumcision Promotion in Ugandan Schools). Results on safe male circumcision have been published previously [15]. In this paper we focus on menstrual practices.

#### Informed consent

Prior to the study, parents/guardians were contacted through the school head-teachers and class teachers who explained and distributed information sheets about the study and consent forms to their students in order to take them home and inform their parents/guardians. To participate in the study, written assent was required from students aged 12–17 years along with their parents/guardians’ consent, and written consent was required from those aged 18 years or older. In boarding schools, school representatives gave consent as the guardians of students. We used a thumb print and signature of an independent witness, in the case of consenting eligible parents/guardians with literacy challenges. A Luganda version of the information sheets and consent forms were available to parents/guardians whenever necessary to facilitate comprehension.

### Data collection

#### Quantitative cross-sectional survey

A cross-sectional study was conducted at each school among all consenting students in Forms 2 and 3 attending school on the day of the survey. Socio-demographic and school absenteeism data were collected from girls and boys, and data on menarche, MHM knowledge and practices from girls. The questionnaires were administered as a self-completed paper form, with a facilitator present to guide students through the questions.

#### Qualitative interviews

Group interviews (GIs) based on a topic guide, were conducted with girls from Forms 2 and 3 (total of eight GIs; one per Form per school). The GIs explored perceptions, taboos, myths and terminologies related to menstruation, menstrual management, school absenteeism, school sanitation facilities and views on proposed interventions. A random sample of 8–12 girls per Form was selected from all girls who were present on the day of the GIs. The GIs were conducted using adolescent-centered participatory methods (i.e. youth facilitation of interviews, and participatory learning and action techniques) [16, 17], and took 60–90 min.

In-depth interviews (IDIs) were conducted to further investigate the issues explored during the GIs. Sixteen girls were randomly selected from the cross-sectional survey (four girls per school, two each from Forms 2 and 3) from those who reported having had at least three menstrual periods. The interviews were conducted at the school by young female interviewers using semi-structured topic guides. They were held in private rooms, lasted 45–60 min and were voice-recorded.

Individual key informant interviews were held with 11 teachers, one municipality official from the Ministry of Education, and one from the Ministry of Health. Each interview took 30–45 min, with the aim of discussing identified strengths, opportunities, challenges and recommendations of existing policies and the school curriculum on puberty. A semi-structured topic guide was used, which contained open-ended and suggested probing questions. The interviews were voice-recorded.

Ten parents (six female, four male) of students were purposively sampled. Topics discussed included parental roles in communicating knowledge, information, attitudes and practices regarding menstruation, to understand their perceptions of MHM-related challenges that girls face in schools and to elicit recommendations for MHM programmes.

#### Diary sub-study

A diary sub-study was conducted to evaluate the acceptability and feasibility of asking girls to self-complete a prospective diary on their menstrual cycles and school attendance. Ten girls who reported having had regular periods over the last 4 months were randomly selected from each school. Participants were asked to self-complete a diary booklet for a six-month period from October 2015 to April 2016 with a box for each day to show whether or not she was menstruating (and if so, whether it was a light, moderate of heavy flow), and whether she attended school for a full-day, half-day or not at all. A female research assistant checked the diaries for completion during unannounced visits to the school (approximately once a month), and assisted the girls to complete these if they were not up-to-date.

#### Water, sanitation and hygiene (WASH) observation checklist

Each school was visited eight times between October 2015 and June 2016. At each visit, research assistants completed a checklist to assess access and state of the following items: number of sanitation facilities by gender, functionality, cleanliness and privacy; facilities for sanitary waste disposal; availability of water, soap and toilet paper.

##### Data management

Paper forms were double-entered onto password-protected Access databases, and transferred to Stata version 14.0 for data cleaning and analysis. The investigators and trained, supervised research assistants kept all research records confidential in lockable cabinets and cupboards.

##### Qualitative data analysis

A thematic content analysis was conducted by a team of four female research assistants (RAs), and a lead analyst. Initially, three interview transcripts were assigned to each research assistant for analysis. Each RA was assigned two additional transcripts that had independently been analysed by one of their colleagues to test inter-rater reliability regarding the themes emerging from the transcripts. This was followed by meetings in which emerging themes and sub-themes were discussed iteratively as further data were collected and analysed, and a coding framework was developed. The framework was further refined and used in the coding and analysis of subsequent scripts. Key themes and subthemes emerging from the data were classified within a matrix.

##### Quantitative data analysis

The cross-sectional questionnaire was analyzed using descriptive statistics to summarize data by school, with comparisons by gender using chi-squared statistics for binary and categorical outcomes, and t-tests for continuous outcomes. Adequate MHM was defined retrospectively from the standard definition from the Joint Monitoring Programme of WHO/UNICEF. This states that “Women and adolescent girls are using a clean menstrual management material to absorb or collect menstrual blood, that can be changed in privacy as often as necessary for the duration of a menstrual period, using soap and water for washing the body as required, and having access to facilities to dispose of used menstrual management materials. They understand the basic facts linked to the menstrual cycle and how to manage it with dignity and without discomfort or fear” [4].

Missing data were not imputed. Factors associated with i) missing five or more days of school in the past month, and ii) at least 1 day of school during the most recent menstrual period, were analysed using multiple logistic regression. School was included a-priori as a fixed effect and other variables associated with the outcome at *p* < 0.10 were included in an initial multivariable model and retained if they were independently associated with the outcome (p < 0.10). Associations between period-days and school attendance in the diary sub-study were analysed using multiple logistic regression, adjusting for within-participant clustering using random-effects.

## Results

### Study participant characteristics

Of the 359 eligible female students (i.e. all those in Forms 2 and 3 attending school on the day of the survey), 352 (98%) gave informed assent and parental consent to participate. All 210 eligible male students gave informed assent and parental consent to participate. The high SES private school was predominantly a boarding school (91.2% of students were boarders), and the other three were mixed day and boarding, with the majority of students being day students (77.4%–96.4%). The mean age was 15.6 years (SD 1.1) for female students (SD 1.1) and 16.4 (SD 1.5) for male students, and was similar by school. Overall, 133 (24.1%) were orphans, including 33 (6.0%) dual (maternal and paternal) orphans. The proportion of dual orphans ranged from zero in the private high SES school to 12.2% in the private low SES school. Overall, 145 (29.6%) of students lived with neither parent. The median time taken walking to school was 30 min for both girls (IQR 20–43) minutes) and boys (IQR 28–58 min). Socio-demographic characteristics of the girls, the focus of this paper, are shown in Table 1.

**Table 1:** Socio-demographic characteristics of girls in the quantitative survey

Characteristic	Government low SES	Government high SES	Private low SES	Private high SES	All schools
N	165	80	55	52	352
Boarding student	6 (3.6%)	21 (26.3%)	10 (18.2%)	48 (92.3%)	84 (24.2%)
Age					
13–14	16 (9.7%)	17 (21.3%)	12 (21.8%)	15 (28.9%)	60 (17.1%)
15	36 (21.8%)	31 (38.8%)	13 (23.6%)	22 (42.3%)	102 (29.0%)
16	59 (35.8%)	25 (31.3%)	22 (40.0%)	13 (25.0%)	119 (33.8%)
17–18	54 (32.7%)	7 (8.8%)	8 (14.6%)	2 (3.8%)	71 (20.2%)
Mean age (SD)	16.0 (1.1)	15.3 (1.0)	15.4 (1.2)	15.1 (0.9)	15.6 (1.1)
Religion					
Catholic	56 (33.9%)	26 (32.5%)	12 (21.8%)	9 (17.3%)	103 (29.3%)
Anglican	35 (21.2%)	26 (32.5%)	13 (23.6%)	17 (32.7%)	13 (23.6%)
Born-again	42 (25.5%)	22 (27.5%)	17 (30.9%)	9 (17.3%)	17 (30.9%)
Muslim	24 (14.6%)	4 (5.0%)	10 (18.2%)	4 (7.7%)	10 (18.2%)
Other	8 (4.9%)	2 (2.5%)	3 (5.5%)	13 (25.0%)	3 (5.5%)
Ethnicity					
Mugandan	75 (45.5%)	31 (39.2%)	36 (65.5%)	15 (28.9%)	157 (44.5%)
Non-Mugandan	84 (50.9%)	45 (57.0%)	16 (29.1%)	23 (44.2%)	168 (47.9%)
Non-Ugandan	6 (3.6%)	2 (2.5%)	3 (5.5%)	14 (26.9%)	25 (7.1%)
Orphan status					
Not an orphan	118 (73.3%)	58 (75.3%)	39 (72.2%)	44 (84.6%)	259 (75.3%)
Maternal	10 (6.2%)	4 (5.2%)	3 (5.6%)	3 (5.8%)	20 (5.8%)
Paternal	24 (14.9%)	7 (9.1%)	5 (9.3%)	5 (9.6%)	41 (11.9%)
Dual orphan	9 (5.6%)	8 (10.4%)	7 (13.0%)	0 (0%)	24 (7.0%)
Maternal education: Primary or below	50 (30.7%)	16 (20.5%)	23 (41.8%)	9 (17.3%)	98 (28.2%)
Paternal education: Primary or below	38 (23.6%)	4 (5.1%)	10 (18.5%)	4 (7.85%)	56 (16.2%)
Live with mother	76 (49.7%)	47 (66.2%)	32 (69.6%)	39 (79.6%)	194 (60.8%)
Live with father	67 (45.3%)	38 (55.9%)	19 (44.2%)	30 (63.8%)	154 (50.3%)
Median household size (IQR)	6 (5–8)	6 (5–9)	6 (5–9)	8 (6–9)	6 (5–9)
Running water inside the house	54 (32.7%)	52 (65.0%)	18 (32.7%)	36 (69.2%)	160 (45.5%)
Toilet/latrine inside the house	65 (40.1%)	34 (47.9%)	13 (23.6%)	35 (68.6%)	147 (43.4%)

### Menstruation perceptions, patterns, symptoms and management

#### Quantitative findings

All but one girl reported having started menstruating (*n* = 351, 99.7%) with median age at menarche 13 years (IQR 13–14 years). Subsequent analyses are among the 351 girls who had started menstruating. The median duration of periods reported in the cross-sectional questionnaire was 4 days (IQR 3–4 days). Physical problems during menstruation were commonly reported with 263 (76.0%) girls reporting “stomach” pain, cramps or bloating, 165 (48.5%) back pain, 134 (39.2%) headaches, 110 (32.0%) irritability/moodiness and 70 (20.4%) genital skin itching. Only 27 girls (7.7%) reported no symptoms during their last menstrual period. In the diary sub-study, girls reported period pains on 68.9% of period-days (252/366 days).

About two-thirds of girls (*n* = 238; 67.8%) disagreed with the statement “Period days are like any other day”, and a similar proportion of boys (*n* = 139, 67.5%) agreed with the statement that girls feel less self-confident during their period days. Overall, 82 girls (23.8%) reported that they had not learnt about periods before their menarche. The main source of information was most commonly the mother (40.6%), followed by peers (24.7%), teachers (14.2%) and other sources (20.5%). When asked whom they did *not* discuss their periods with, about half the girls said their fathers (52.4%).

During their last period, 305 (86.9%) girls reported using disposable manufactured sanitary pads (Table 2), 44 (12.5%) used a locally-manufactured re-usable pad (e.g. AFRIpads), and about a third of girls (32.2%) used a combination of methods. Only 4 girls (1.1%) reported adequate MHM at their last period using four components of the standard definition that were asked about directly:

i. Proportion who only used manufactured products (which may by locally-made re-usable pads (e.g. AFRIpads, disposable manufactured pads (e.g. Always) or tampons) during their last period: 228/351 = 35.0%
ii. Proportion who use disposable pads or tampons and dispose of them in a bin or incinerator: 104/293 = 35.5%
iii. Proportion who report always having access to water and soap at school: 50/351 = 14.2%
iv. Proportion of girls who say they do not feel anxious about their next period: 54/351 = 15.1%

**Table 2:** Management of menstruation among 351 girls who had passed menarche

Characteristic	Government low SES	Government high SES	Private low SES	Private high SES	All schools
N	165	80	54	52	351
Protection used during last period					
Disposable manufactured pads	142 (86.1%)	69 (86.3%)	47 (85.5%)	47 (90.4%)	305 (86.7%)
Tampons	7 (4.2%)	5 (6.3%)	1 (1.8%)	3 (5.7%)	16 (4.6%)
Locally-made manufactured re-usable pads	27 (16.4%)	3 (3.8%)	10 (18.2%)	4 (7.7%)	44 (12.5%)
Old clothes	31 (18.8%)	5 (6.3%)	7 (12.7%)	1 (1.9%)	44 (12.5%)
Toilet paper	20 (12.1%)	6 (7.5%)	6 (10.9%)	1 (1.9%)	33 (9.4%)
Cotton wool	26 (15.8%)	6 (7.5%)	8 (14.6%)	1 (1.9%)	41 (11.7%)
Underwear only	16 (9.7%)	6 (6.3%)	8 (14.6%)	1 (1.9%)	30 (8.5%)
Times changed protection per 24 h during last period					
Once	5 (3.1%)	8 (10.3%)	4 (7.4%)	1 (2.0%)	18 (3.3%)
Twice	45 (28.0%)	31 (39.7%)	23 (42.6%)	12 (24.5%)	111 (32.5%)
Three times	82 (50.9%)	34 (43.6%)	16 (29.6%)	19 (38.8%)	151 (44.2%)
Four times	20 (12.4%)	5 (6.4%)	6 (11.1%)	9 (18.4%)	40 (11.7%)
> =5 times	9 (5.6%)	0 (0%)	5 (9.3%)	8 (16.3%)	22 (6.4%)
Ever had menstrual accident with blood leaking to clothes					
Ever^a^	114 (69.1%)	55 (68.8%)	30 (55.6%)	26 (51.0%)	225 (64.3%)
During last period	32 (19.5%)	16 (20.3%)	6 (11.1%)	10 (22.2%)	64 (18.7%)
Re-usable/washable protection (i.e. Clothes, reusable pads or other washable protection)					
Use re-usable/washable protection^b^	80 (52.0%)	14 (20.3%)	16 (29.6%)	7 (17.5%)	117 (36.9%)
Wash material water & soap^b^	75 (93.8%)	13 (92.9%)	15 (93.8%)	6 (85.7%)	109 (93.2%)
Share material^b,c^	5 (6.7%)	3 (23.1%)	4 (25.0%)	0 (0%)	12 (10.3%)
Dry material outside ^b,d^	20 (25.0%)	3 (21.4%)	5 (31.3%)	5 (71.4%)	41 (23.4%)
Disposable protection					
Ever used disposable pads or tampons^e^	149 (90.3%)	68 (85.0%)	51 (94.4%)	45 (94.4%)	313 (89.2%)
Use them every day of period^a, f^	109 (73.7%)	56 (82.4%)	35 (70.0%)	39 (92.3%)	239 (77.6%)
Would try a re-usable pad	127 (85.2%)	39 (59.1%)	37 (72.6%)	31 (70.5%)	234 (75.5%)
Reason for choosing disposable pads^c^					
Less worry about leaks^g^	124 (84.4%)	48 (75.0%)	26 (51.0%)	35 (79.6%)	233 (76.1%)
More comfortable^h^	134 (90.4%)	57 (86.4%)	41 (80.4%)	38 (86.4%)	270 (87.4%)
Easier disposable^i^	111 (74.5%)	53 (77.9%)	32 (62.8%)	31 (68.9%)	227 (72.5%)
No need to wash/dry^j^	125 (86.2%)	58 (86.6%)	41 (80.4%)	33 (76.7%)	257 (84.0%)
More modern^k^	48 (33.8%)	16 (25.8)	12 (23.5%)	13 (32.5%)	89 (30.17%)

^a^Missing data for 3 participants

^b^In general, not in the last period. This includes re-usable pads, old clothes, knickers

^c^Missing data for 6 participants

^d^Missing data for 1 participant

^e^Missing for 4 participants

^f^Among those who reported ever using disposable pads or tampons

^g^Missing for 7 participants

^h^Missing for 4 participants

^i^Missing for 11 participants

^j^Missing for 7 participants

^k^Cloths are viewed as traditional and associated with low income. Missing for 18 participants

Multiple reasons were given for choosing disposable protection, with a majority reporting they chose these because it reduced concern about leaks (74.4%), were more comfortable (86.3%), were easier to dispose of (72.5%) and there was no need to wash or dry them (92.1%). Overall, 239 girls (77.6%) of girls who reported ever using disposable absorbents reported being able to afford to use them every day of each period. However, most girls who currently used a disposable manufactured pad said they would be interested in trying a locally-made pad (*n* = 234; 75.5%) with the main reason being if it was cheaper (*n* = 114; 45.1%) or re-usable (*n* = 86; 34.0%). When asked about the importance of attributes of sanitary protection, 316 (90.3%) reported disposable protection as important or very important, 170 (48.9%) washable protection as important or very important.

Menstrual accidents were commonly reported. Most girls (81.9%) reported changing their protection fewer than 4 times in a 24-h period, and almost two-thirds (*n* = 225; 64.3%) reported ever having experienced leakage of menstrual blood on to their outer clothes, with 18.7% reporting having stained their outer clothes during their most recent menstrual period (Table 2). The prevalence of this was similar by age (18.2% vs 19.4% in those aged 16–18 vs 13–15 years, respectively). Over a third of boys (*n* = 76, 37.1%) reported having seen a girl have a menstrual accident.

#### Qualitative findings

Menstrual pain was a key concern of girls, and management of menstruation was affected by their limited access to analgesics, and the widespread belief that the use of analgesics would be detrimental to health.

*“They told me when you are in your periods and you get cramps, do not use Paracetamol; that it is not good for our future health; that instead you should put warm water in a plastic bottle and press it against the lower abdomen”.* (GI 02 participant at low SES private school)

*“People say that when you take painkillers it will not be easy to give birth, that your blood flow reduces so the blood which was meant to come out remains inside and rots”.* (GI 11 participant at high SES private school)

The IDIs with girls indicated lack of knowledge about menstruation and its management prior to menarche, and that menarche caused anxiety:

“*I was so scared actually I thought it was a disease that had attacked me. I thought I was going to lose my life at any moment because it was really scary to start bleeding from down there. I thought I was going to die”.* (IDI participant 05 at high SES private school)

*“I was embarrassed, and I did not know what menstruation was when I started, so I was confused and did not know what it really meant. I felt embarrassed to tell other people, and to make myself comfortable I decided to keep it a secret.”* (IDI participant 05 at high SES private school)

Findings from IDIs with parents also indicated their awareness of the girls’ limited knowledge of menstruation and MHM.

*“I remember before they went back for this term she was telling the mother as I was listening. [She was explaining that] when she experienced menarche she did not know [what was going on] so she told the head of women teachers that she was seeing funny things, and that she advised her”.* (Guardian 07, Male)

Lack of knowledge of menstruation was indicated to be largely due to guardians’ (parents’ or carers’) disengagement regarding puberty, menstruation and sexuality. For example when girls told guardians about their menarche, this did not always lead to a discussion of menstruation.

*“I went to her, she was sitting. Then I said “Grandmother, blood is coming!” Then she asked, “Where?” I said “in my private parts!” Then she said “okay, you go in the bathroom, let me just go to the shop and come back.” I didn’t know why she was going there, she just told me that I should go in the shower … she never told me what she was going to do from there”.* (IDI participant 06 at low SES public school)

These findings are consistent with those from IDIs with the girls’ parents/guardians who acknowledged disengagement with girls, especially on matters of menstruation. It was clear from their perspectives that cultural norms played an important role in undermining parent-girl dialogue on menstruation and its management.

*“I would love it so much to engage my children but am shy to talk about those issues…. That is why if a teacher is in a position to do it, or if I can get a friend of mine to go ahead and talk to my child about those issues [I would welcome the opportunity]”.* (Guardian 01, Female)

The lack of knowledge and confidence in effective MHM was present for both newly-menstruating and experienced girls. Confidence in managing menstruation was undermined by both disengagement between girls and their guardian and by lack of adequate protection methods. Lack of access to protection methods was mentioned by the girls and confirmed by the guardians.

*“Speaking of those girls who don’t have materials like pads, you find that the dad is poor, the mom is poor, some have step-moms and there is no provision of that [pads]…. So they do not get the basic things they need when they are at school”.* (Guardian 09, Male)

While in some cases girls did not use any absorbent material, most indicated having used pieces of old cloth, underwear, cotton and handkerchiefs because they could not afford to solely use disposable sanitary pads.

*“On the first day I did not use anything at all and on the second day my mum did not have money to buy me pads so she told me to get a piece of cloth that I no longer use and then sow it around the knickers and she told me that it should be big for the blood not to go through. But she told me not to use cotton or toilet paper; that it is dangerous. I went to the bedroom and started looking for an old bed sheet that we no longer use and used that”.* (IDI participant 04 at high SES public school)

### School attendance and menstruation

#### Quantitative findings

##### Cross-sectional study

Both boys and girls reported having missed a median of 2 days of school in the last month (IQR 0–5). The majority of students (59.9% of girls and 64.7% of boys) reported having missed at least 1 day of school in the past month, and almost one third (28.2% of girls and 30.9% of boys) reported missing ≥5 days of school in the past month. The most common reason given for missing school differed by gender, with inability to pay school fees more commonly reported for boys (44.1% of girls and 62.6% of boys who missed at least 1 day) and “sickness” for girls (44.6% of girls and 19.1% of boys, *p* < 0.001). 9.5% of girls said that in general, they didn’t attend school during menstruation, and 17.3% gave menstruation as the main reason for them missing school (29.3% of those who missed at least 1 day in the last month).

Multivariable logistic regression showed that, among girls, missing ≥5 days of school in the past month was independently associated with older age (aOR = 2.53, 95%CI 1.08,5.96 for those aged 15–17 vs 12–14), religion (aOR = 0.35, 95%CI 0.13,0.99 for Muslim vs Catholic), ethnicity (aOR = 2.18, 95%CI 1.24,3.81 for non-Muganda vs Muganda), lower SES (aOR = 2.34, 95%CI 1.34,4.11) and being a maternal orphan vs not being an orphan (aOR = 2.08, 95%CI 0.98, 4.43HM).

Overall, 69 girls (19.8%) reported missing at least 1 day of school during their last period and 61 girls (17.3%) reported missing school in the last 30 days due to menstruation – but 42 girls did not answer these two questions consistently. There was little evidence that the proportion of girls missing at least 1 day of school during their last period was associated with the specific school that they attended (ranging from 15.4% in the high SES private school to 22.0% in the low SES government school). The most commonly reported reasons for missing school during menstruation were stomach or back pain (92.5%), feeling generally unwell (60.0%), fear of leaking blood (38.5%), and lack of privacy for changing (38.5%). When asked to name the main reason, the majority said pain (85.7%). Among those who reported missing school during menstruation, fear of leaking blood was slightly more common among girls who reported changing protection 4 or more times per day (9/17 = 52.9%) than among those who changed less frequently (16/52; *p* = 0.10). In multivariable analyses, missing at least 1 day of school during the last period was independently associated with older age and non-Muganda ethnicity (Table 3). After adjusting for these socio-demographic factors and school, reported missing at least 1 day of school during the last period was associated with having to change protection more regularly, heavy blood flow, and with symptoms (headache, stomach pain, backache) (Table 3). The association with changing protection frequently became non-significant after adjustment for reported blood flow (aOR = 1.71, 95%CI 1.21–4.24) but was not confounded by reported symptoms.

**Table 3:** Factors associated with missing at least one day of school due to menstruation in the past month

	N	Number missing at least one day of school due to menstruation (%)^a^	Adjusted odds ratio (95%CI)
Total	351	69 (19.8%)	
Age			*P*-value for trend < 0.001
13–14	58	5 (8.6%)	1
15	102	15 (14.7%)	1.88 (0.64–5.56)
16	117	27 (23.1%)	3.04 (1.07–8.60)
17–18	71	22 (31.0%)	4.72 (1.56–14.25)
Ethnicity			P = 0.01
Mugandan	155	20 (12.9%)	1
Non-Mugandan	166	41 (24.7%)	2.26 (1.23–4.15)
Non-Ugandan	25	7 (28.0%)	2.97 (1.00–8.75)
Times changed absorbent per 24 h during last period^b^			*P* = 0.03
< =3 times	277	50 (18.1%)	1
> =4 times^c^	62	18 (29.0%)	2.08 (1.06–4.10)
Use disposable pads for each day of period^2^			*P* = 0.22
No	79	21 (26.6%)	1
Yes	262	48 (18.3%)	0.68 (0.36–1.26)
Amount of blood lost on heaviest day of period^2^			
Little	40	3 (7.5%)	0.36 (0.10–1.28)
Average	213	34 (16.0%)	1
Very much	89	30 (33.7%)	2.45 (1.34–4.48)
Symptoms during last period			
Headache^b^			*P* = 0.01
No	206	31 (15.1%)	1
Yes	132	35 (26.5%)	2.15 (1.20–3.86)
Stomach pain ^b^			*P* = 0.10
No	83	10 (12.1%)	1
Yes	260	58 (22.3%)	1.89 (0.89–4.04)
Back pain^b^			*P* = 0.06
No	174	24 (13.8%)	1
Yes	163	43 (26.4%)	1.75 (0.97–3.14)

^a^Excluding 3 girls with missing outcome data

^b^Adjusted for age, ethnicity and school

^c^The recommended number of changes would be 5 (one every 4 h during the day, plus one at night) but as only 6% of girls changed this frequently we used a cutoff of 4 or more changes in 24 h

##### Diary sub-study

An estimate of the relative frequency of school absenteeism on days with periods and without periods was obtained from the diary data. This included data on 366 period-days and 2271 non-period days from 39 girls, with an average of 80 entries per girl over two terms. Data on periods were missing for only 12 school days (0.45%). On non-period days, 6.5% (*n* = 147) days of school were reported to have been missed, compared with 28.4% of period-days (adjusted OR = 5.99; 95%CI 4.4,8.2). There was no evidence of effect modification by type of school (high vs low SES; *p* = 0.40). From this, the excess risk of missing school on a period-day is 21.9%. Assuming that an average of 2.86 period-days per month are school days (based on 5 school days a week) and with 9 school-months per year, this means that a girl would miss an average of 6 school days per year specifically because of menstruation.

##### Observation of WASH facilities

Direct observation of the WASH facilities showed that pour-flush toilets were available in the two high SES schools but only pit latrines in the low SES schools. The majority of pour-flush toilets had locks (16/20) but only 12/23 of the pit latrines did. None of the schools had toilet paper or soap available. All schools had disposal bins for sanitary waste available, except the private low SES school.

#### Qualitative findings

While key informants cited a variety of factors undermining school attendance (including long distance to school, competing domestic chores and poor oversight by parents), menstruation and poverty were reported to be the most important. All of the 14 key informants cited menstruation as an impediment to school attendance. Similarly, nine key informants cited poverty as a barrier to school attendance, and this was reflected in the lack of school materials, such as textbooks. The critical role of menstruation and poverty in influencing school attendance was also reflected in the IDIs with parents. In the case of poverty, a student who was asked to go home and return only when they had procured the required textbook might miss several schooldays while they raised the money to purchase the book. Poverty also resulted in some students failing to raise money for their school fees, which often led to their suspension from school until the fees were paid. This was particularly common in private schools.

During the IDIs with girls, menstruation was invariably cited as the key factor explaining girls’ absence from school. The main reasons given for why menstruation kept girls away from school included pain, lack of access to protection methods, and lack of privacy for MHM at school.

*“If I wake up in the morning when I am having my menstrual period, I never go to school because I get terrible cramps …. I have not been coming to school when I am menstruating”* (Group Interview 08, participant at low SES private school).

*“Sometimes when … mother has no money to buy material [sanitary pads] I don’t bother coming”* [to school] (Group Interview 07 participant at low SES public school).

*“Yes, someone can peep at you. When boys are revising [reading books] from that side you actually don’t feel safe. Boys can even see you from their toilets”* (Group Interview 13 participant at high SES public school).

The fear of having a menstrual accident and subsequent humiliation from the boys was also reported to affect school attendance. Several girls echoed the following sentiments from an IDI participant:

*“The boys laugh at us … when you soil your uniform and you aren’t aware. Instead of them letting you know, they call their friends to tell them how you have soiled your uniform”* (IDI 03 participant at low SES public school).

Notably, girls’ education was not only undermined by absence from school but also absence from class – especially among boarders. Restrictions on use of washrooms while the class was in session was also reported to contribute to girls staying away from school and from class during menstruation.

*“We are supposed to go to the latrines at only break time, lunch time and evening time so we are not allowed to be there when its class time because that is a time when she [toilet cleaner] is cleaning; she can only let you in if you are having a serious menstrual period”* (IDI 04 participant at high SES public school).

School attendance was also affected by girls’ feelings of embarrassment and being dirty, and a keen awareness of what boys and teachers think about girls who are menstruating.

*“I felt like, oh no… I didn’t want to stand up in class. My friends told me to go for lunch (with them). I told them I didn’t want to because I was having my periods …. I thought, like, I didn’t want anyone to come near me. I thought, like, they will get to know. Maybe I’m smelling ….”* (IDI 10 participant at high SES public school).

The stigma of menstruation posed barriers to their learning even among those who had access to good menstrual hygiene materials. This stigma resulted in avoidance of teasing from the boys by not attending class or school, but also avoiding gender insensitivity of male teachers, and other school staff (such as toilet cleaners), as well as the gender insensitive physical infrastructure at schools.

*“Some male teachers just start harassing girls. Like if a girl gets her menstruation when she was not prepared and then explains to the teacher, he can be, like, “You are stupid”…”* (IDI participant 04).

*“In secondary it is not bad, but in primary if a teacher saw you tying a sweater around your waist he could call you and ask why you have done this even when he knows [that you are covering yourself to avoid a possible embarrassment of a blood-stained dress]”* (IDI participant 02).

### Improving menstrual management in schools

In the cross-sectional questionnaire, girls were asked to rank 8 suggestions for improving MHM at school. The highest-ranking suggestions addressed mainly ‘hardware’ issues, with two-thirds of girls (*n* = 236, 67.1%) including in their top three suggestions that schools should stock sanitary towels, and half (*n* = 184; 52.4%) recommending that plenty of water was made available (52.6%). Other common recommendations were to stock analgesics (42.3%), ensure that girls who are menstruating do not receive corporal punishment (37.8%) and that girls’ toilets allowed privacy from boys (33.6%). Suggestions that appeared less frequently in the top three suggestions included allowing girls to remain seated whilst asking questions (25.3%) and ensuring there were appropriate disposable bins for disposing of sanitary pads (25.6%).

The findings from the qualitative data were consistent with those from the cross-sectional questionnaire. The main recommendations from participants in the group interviews, IDI and key informant interviews were related to the need for improving the supply of MHM materials, and the facilities at school to make it easier for girls to clean themselves and dispose of used pads in a way that assures their privacy. One of the most frequent recommendations was for the availability of MHM materials including sanitary pads and analgesics.

*“Organizations should come up and give out materials for girls during menstruation. For example pads and knickers …. [Also] pain killers should be provided to help those who get painful cramps and periods”* (Group Interview 02 participants at low SES private school)

*“They should give [us] enough pads, underwear and at least buy for [us] some painkillers, because some schools have no sick bays like us here. Sometimes you go there and there are no painkillers….”* (IDI participant 15 at high SES private school)

Improvement of facilities at school was also frequently cited. Participants particularly pointed out the need for a steady supply of water and soap at school.

*“They should put water and soap in our toilets because most of the time you find when our tap is locked even when you want to clean yourself up you cannot”* (IDI participant 02 at low SES private school)

Students also repeatedly recommended that schools address their concerns about the lack of privacy, especially during menstruation.

*“Provide bathrooms where girls can go to clean up because [boy] students can come and pull the door when you are still cleaning up yourself”* (Group Interview 13 participant at high SES public school)

Other recommendations included ‘software’ issues such as the need to sensitize male teachers and male students so they can understand the challenges female students go through when menstruating, in the hope that they can treat them less harshly, as well as the need to identify senior women teachers who would act as point persons for the students in case they needed pads or counselling.

## Discussion

Management of menstruation is challenging among secondary students in peri-urban Uganda, for both newly menstruating and experienced girls, and involves psychosocial and physical challenges. To date, most studies have focused on MHM among rural primary school students, and this study adds to the literature in focusing on secondary students, and those in a peri-urban rather than rural setting.

The qualitative data and prospectively collected diary data showed clear evidence that menstruation was associated with school absenteeism. For example, in the diary study, girls reported missing school four times more frequently during their period than when not menstruating. The qualitative findings support previous studies in LMIC showing that menstruation is a cause of distress to many girls, and a barrier to school attendance [3, 18], including a recent qualitative study among school girls in rural Uganda [19]. There were also reports of an association of menstruation and school absenteeism in the cross-sectional survey with 10% of girls saying that in general they didn’t attend school during menstruation, and about 20% reporting missing at least 1 day of school during their last period. However, there were inconsistent reports of school absenteeism due to menstruation in the quantitative cross-sectional survey. Most quantitative studies have not found an association between menstruation and school attendance [12, 13, 20, 21], and a recent systematic review identified only 3 intervention trials (in Nepal [12], Ghana [11] and Kenya [14] respectively), which assessed school attendance as an outcome. The two African studies found a moderate non-significant effect (standardized mean difference = 0.49, 95%CI -0.13,0.11), but no association was seen in Nepal where the overall school attendance was very high [10].

Challenges of assessing school attendance through retrospective quantitative surveys include possible under-reporting due to social desirability bias not to report school absenteeism, or to name menstruation as a reason for this. For example, a recent study in Kenya asked girls to complete a monthly calendar to document menstruation and school attendance (with follow-up visits by nurses to check completion) but found that school absenteeism was rarely reported, precluding analysis [13]. In our study, a young adult female research assistant collected the diaries from the girls, and we hypothesize that this reduced under-reporting of school absenteeism compared with data collected by teachers or more formal research staff. Further, school absenteeism is difficult to measure accurately as school registers are frequently not accurate. It is possible that girls exaggerate the association in the qualitative interviews and diaries although what motivation they might have for doing this is not clear. Based on the internal consistency of the qualitative findings and the methodological strengths inherent in the diary method where data are recorded on a daily basis, together with inconsistent reporting from the cross-sectional questionnaire, we conclude that in our setting, with follow-up visits by a trusted research assistant, the prospectively collected diary data is more likely to be accurate. A limitation of our study was that it was not designed to directly compare the reported school absence due to menstruation from diaries and the cross-sectional survey, as the diary sub-study involved detailed information from a small sample of girls and focused on feasibility of using this method. Further work on the accuracy of this method for collecting school attendance and menstruation data is needed, including examination of how the diaries are administered and whether they are perceived as being independent of school teachers. Further work is needed to triangulate measures of school attendance including spot checks, use of routine school registers and diaries kept by students.

It is notable that our study found similar levels of absenteeism reported by boys and girls, and this has been seen in other settings [20, 22, 23]. This is often taken to argue against an association of menstruation with school absenteeism, but it may reflect other differences in the reasons that girls and boys miss school. For example in our study, the primary reason that boys gave for missing school was a lack of school fees, whereas girls were more likely to report being “sick”. The discrepancy between this widespread observation that reported school absenteeism rates are similar in boys and girls and the clear results of the qualitative research suggesting that school absenteeism is related to menstruation for at least a proportion of girls is striking and merits further research.

The methods of managing menstruation seen in this peri-urban secondary school population differed from that in rural Uganda [24, 25], where most students reported primarily using re-usable pads, for example Afripads and Makapads which are Ugandan-made, low-cost pads sold or given directly to schoolgirls by non-governmental organisations [26]. For example, among 10–19 year old girls in rural schools in Kamuli district, Eastern Uganda, only 9% of girls reported using disposable pads as their main protection [25]. In our peri-urban setting, even though the majority of girls reported being able to afford disposable pads for every day of their period, over 80% reported changing the absorbent fewer than four times a day, and 20% reported leaking blood to their outer garments during their most recent menstrual period. It is not clear whether this was due to being unprepared for the start of the period, or due to inadequate management during the period. A limitation of our study was that we did not include questions to address all components of the standard definition of adequate MHM. However, it was striking that only 4 girls (1.1%) reported having even four components of the definition (absorbent material, adequate disposal, access to water and soap, and lack of anxiety about their next period). This highlights the need for interventions to improve MHM even in relatively affluent settings.

Lack of knowledge about MHM was a source of anxiety among the girls in this study, and this can lead to inadequate menstrual hygiene management (e.g. to predict onset of next period) [19, 27, 28]. Confidence in managing menstruation was undermined by disengagement of guardians, and lack of access to adequate protection methods, due mainly to poverty. Further work should explore whether improved puberty education and the use of a diary decreases leaking by improving prediction of the next due date. The role of the parents was clear from both qualitative and quantitative studies with mothers being the primary source of information on menstruation, and higher rates of menstruation-related absenteeism among maternal orphans. This, together with the stigma and fear of teasing from boys, teachers and other school staff highlights the importance of improving knowledge and discussion of puberty and menstruation in the schools, families and community. Few other studies have examined the relationship between orphanhood and menstruation-related absenteeism, although there is a large literature on orphanhood and school attendance more generally. A comprehensive review of population-based surveys in 40 sub-Saharan African countries showed that orphans had consistently lower levels of education than non-orphans [29]. A study from Kenya found that orphans and vulnerable children (OVCs) had better access to sanitary pads than non-orphans as they received sponsorships that paid for supplies, alongside school fees, meals and uniforms [30]. Further to this, a study in Tanzania and Uganda found that, in Uganda, school absenteeism was highest among non-supported OVCs, and lowest in supported OVCs, with non-orphans in between. The results differed in Tanzania with the lowest rates among non-supported OVCs [23].

The study also highlighted that girls’ education is not only undermined by absence from school but also absence from class. This especially applied to those who were in boarding school where girls missed class for the same reasons that their counterparts in day school stayed away from school, either by staying in their dormitory or going to the nurse’s office. The stigma of menstruation can pose barriers to their learning due to pain, or due to the fear of leakage or teasing from boys or teachers and other school staff (such as toilet cleaners). A limitation of our study was that we did not capture school engagement and performance, and this will be important in future studies, for example using school examination data. Another limitation was the relatively small size we had, particular within schools, as this was a descriptive study enrolling all pupils in the relevant classes for future interventions. There was little missing data on periods in the diaries, which we attribute to the checking of diaries by the research assistant at unannounced monthly visits. It is possible that the girls completed the diaries to bias towards an association of periods with missing school, but unlikely as they were not aware that we were particularly interested in this association and they were keen to use the diaries to keep track of their menstruation cycles.

Among the girls who reported missing school during menstruation, the main reasons were pain, fear of leaking and lack of privacy. Fear of leaking was slightly more common among those who changed protection frequently, suggesting that the fear may be due to anxiety about leakage or heavier flow, rather than inability to afford adequate protection. Further, the observational spot checks showed that none of the schools had toilet paper or soap available and half of the pit latrines did not have door locks that could offer privacy to the girls. The main suggestions from study participants to mitigate menstruation-related absence reflected these challenges, and based on this and the findings from the qualitative and quantitative studies, we propose that an effective and cost-effective intervention package to improve menstrual hygiene management in this setting should take a comprehensive approach including both ‘software’ elements such as improving knowledge and attitudes towards menstrual health, as well as ‘hardware’. This would include i) improved training of teachers to provide puberty education, ii) provision of analgesics and iii) improved WASH facilities (e.g. including installing locks on the toilet and latrine doors, repairing broken or incomplete doors, providing bins for pads, toilet paper in a holder, and a soap dispenser). Tools to improve tracking the menstrual cycle (such as the diary), and introduction to new methods such as the re-usable menstrual cup, would also be potentially effective and acceptable.

## Conclusion

Menstruation was strongly associated with school attendance in this peri-urban setting, and that there is an unmet need to investigate interventions for girls, teachers and parents which address both the knowledge and psychosocial aspects of menstruation (self-confidence, attitudes) and the physical aspects (management of pain, use of appropriate materials to eliminate leakage of menstrual blood, improved WASH facilities).

## Abbreviations

CI: Confidence interval
GI: Group interviews
IDI: In-depth interview
LMIC: Low and middle income countries
MENISCUS: Menstrual hygiene and safe male circumcision promotion in ugandan schools
MHM: Menstrual hygiene management
OR: Odds ratio
OVC: Orphans and vulnerable children
SES: Socio-economic status
USE: Universal secondary education
WASH: Water, sanitation and hygiene
